# Atypical Nail Manifestations in Kawasaki Disease: Orange-Brown Chromonychia in a Three-Year-Old Boy

**DOI:** 10.7759/cureus.91377

**Published:** 2025-09-01

**Authors:** Khalil Elouadghiri Fouad, Ikram El Hachmi, Anane Sara, Aziza Elouali, Maria Rkain, Abdeladim Babakhouya

**Affiliations:** 1 Department of Pediatrics, Faculty of Medicine and Pharmacy, Mohamed First University, Oujda, MAR; 2 Department of Pediatrics, Mohammed VI University Hospital Center, Oujda, MAR

**Keywords:** early diagnosis, kawasaki disease, nail abnormalities, orange–brown chromonychia, pediatric vasculitis

## Abstract

Kawasaki disease (KD) is an acute childhood vasculitis characterized by a well-established set of clinical signs. Among the mucocutaneous manifestations, nail abnormalities are mainly observed during the convalescent phase. However, orange-brown chromonychia is a rare and early finding, still poorly documented, that could serve as an additional diagnostic clue. We report the case of a three-year-old boy admitted for persistent fever lasting seven days, presenting with the classic clinical criteria for KD, along with orange-brown chromonychia appearing on the seventh day of fever. Laboratory investigations revealed a marked inflammatory syndrome without myocardial involvement. The outcome was favorable under high-dose intravenous immunoglobulins and aspirin, with progressive resolution of the chromonychia within four weeks. A literature review identified several similar cases, mainly reported in Asia, the Americas, and Europe. To our knowledge, no cases have yet been described in Africa. Analysis of available data suggests that this nail abnormality generally occurs between the fifth and tenth days of the disease and resolves spontaneously. Its mechanism remains uncertain but may involve periungual vascular alterations. This observation highlights the importance of recognizing chromonychia as an early manifestation of KD, particularly in atypical forms.

## Introduction

Kawasaki disease (KD) is an acute systemic vasculitis of medium-sized vessels that predominantly affects children under five years of age [1,2]. It is the leading cause of acquired heart disease in this age group in developed countries [1]. The diagnosis is primarily based on well-defined clinical criteria, including prolonged fever associated with several mucocutaneous and lymphadenopathic signs [3]. However, atypical or ancillary clinical manifestations, although not included in the classical diagnostic criteria, are sometimes observed and may broaden the understanding of the disease’s phenotypic spectrum. Among these, nail abnormalities have been reported, with transverse leukonychia and Beau’s lines being the most common [4]. Orange-brown transverse chromonychia has also been occasionally described in the literature [5]. This subtle sign, usually appearing during the subacute phase of the disease, remains rare and poorly documented. Its true prevalence is uncertain, partly due to its transient nature and the lack of recognition as a clinically significant feature.

We report here the case of a child with KD presenting with characteristic bilateral nail involvement. This case underscores the value of careful nail examination as part of the clinical follow-up in KD, especially in children with atypical or incomplete forms.

## Case presentation

A three-year-old Moroccan boy, with no significant past medical history and no recent medication use, was admitted to our pediatric department for persistent fever lasting seven days. Upon admission, the child was febrile at 39.5 °C, markedly irritable, and tachycardic at 128 beats per minute. Blood pressure was within normal limits for age and height. The respiratory examination revealed eupnea with a respiratory rate of 20 breaths per minute and an oxygen saturation of 97% on room air. Anthropometric assessment showed a weight of 16 kg and a height of 112 cm, indicating adequate nutritional status.

**Figure 1:**
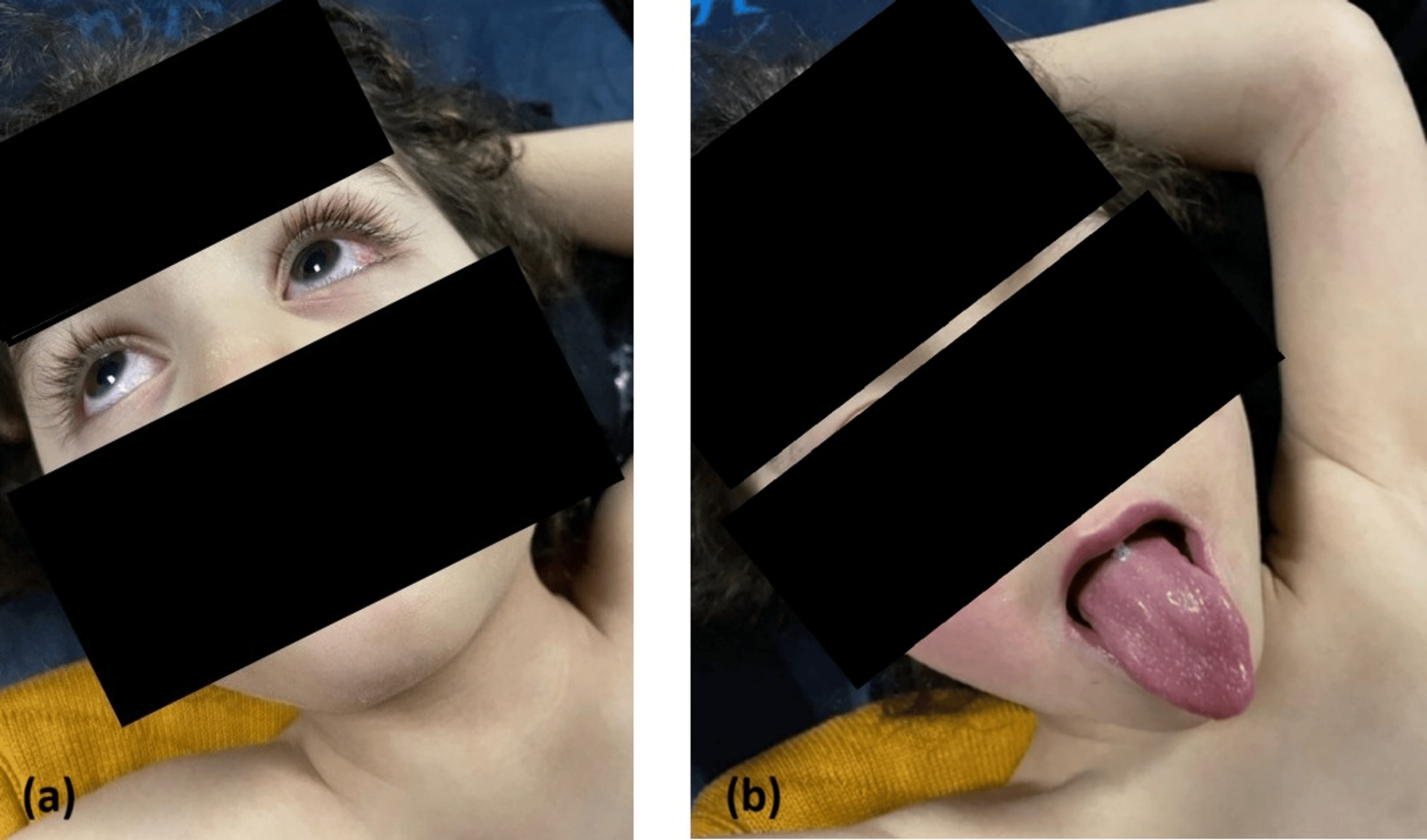
(a) Bilateral non-purulent conjunctivitis observed during the acute phase of Kawasaki disease; (b) Typical “strawberry tongue” appearance (lingual erythema with papillary hypertrophy) during the acute phase of Kawasaki disease

**Figure 2:**
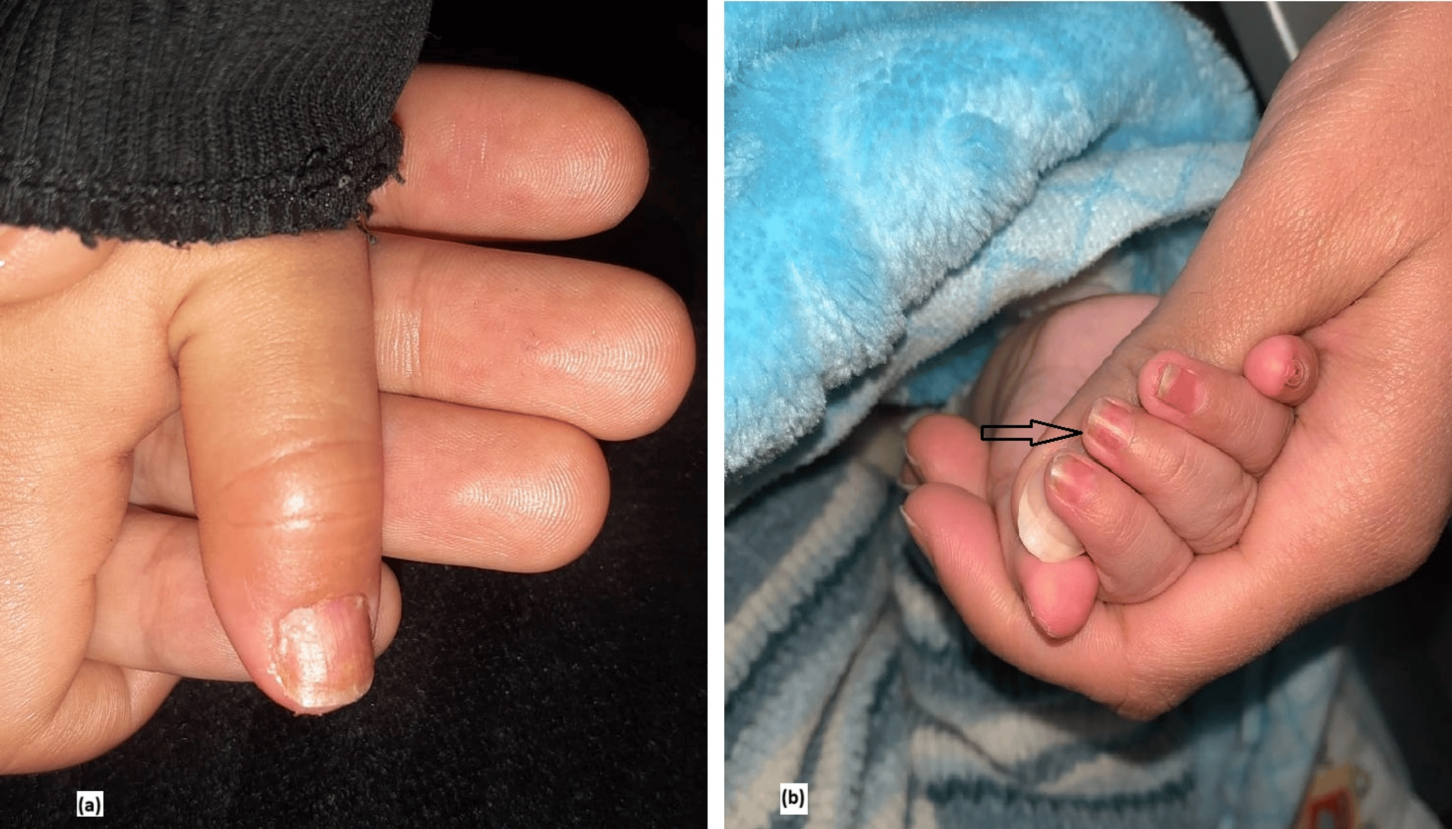
Distal chromonychia in Kawasaki disease: (a) Close-up view of the thumb; (b) View of the other fingers of the hand (black arrow), also affected

Clinical examination revealed bilateral non-purulent conjunctivitis, moderate cheilitis associated with a “strawberry tongue,” and extremity edema with early digital desquamation, particularly of the right thumb. A mobile left cervical lymphadenopathy measuring approximately 3 cm was also noted, along with a maculo-erythematous rash localized to the face. A distinctive finding during examination was the presence of transverse orange-brown discoloration of the fingernails and toenails, which had appeared on the day of admission. This presentation, suggestive of bilateral chromonychia, represented an unusual sign in this clinical context (Figures 1, 2).

Given the combination of five major criteria (prolonged fever, conjunctivitis, oral enanthem, polymorphous rash, cervical lymphadenopathy), the diagnosis of classic KD was established. The main alternative etiologies of chromonychia, including fungal infections, bacterial colonization, drug- or topical-induced nail discoloration, trauma, and systemic disorders of hepatic or renal origin, were excluded. This exclusion was supported by the absence of recent drug or topical exposure, the lack of clinical features suggestive of systemic disease, normal hepatic and renal function tests, and the absence of local signs of bacterial colonization such as periungual inflammation, pain, suppuration, or the characteristic greenish discoloration. In this context, chromonychia was therefore attributed to KD.

Initial laboratory evaluation revealed a marked inflammatory syndrome associated with moderate inflammatory anemia, with no biological evidence of myocardial involvement (Table 1).

**Table 1:** Laboratory results at admission CRP: C-Reactive Protein; MCV: Mean Corpuscular Volume; MCH: Mean Corpuscular Hemoglobin; ESR: Erythrocyte Sedimentation Rate; AST: Aspartate Aminotransferase; ALT: Alanine Aminotransferase; NT-proBNP: N-Terminal pro-B-type Natriuretic Peptide

Parameter	Result	Pediatric reference range
Hemoglobin (Hb)	11.1 g/dL	11.5 – 14.5
MCV	76 fL	77 – 95
MCH	26 pg	26 – 33
White blood cells (WBC)	13,100/mm³	5,000 – 14,000
– Neutrophils	7,470/mm³	1,500 – 7,500
– Lymphocytes	3,800/mm³	1,500 – 4,500
Platelets (PLT)	544,000/mm³	150,000 – 400,000
CRP	62 mg/L	< 5
ESR	67 mm (1st h)	< 20
Fibrinogen	4.2 g/L	2 – 4
Ferritin	190 ng/mL	20 – 200
Sodium (Na⁺)	136 mmol/L	135 – 145
Potassium (K⁺)	4.5 mmol/L	3.5 – 5.0
Total calcium (Ca²⁺)	97 mg/L	90 – 105
Albumin	37 g/L	35 – 50
Total proteins	64 g/L	60 – 80
AST	26 U/L	< 45
ALT	20 U/L	< 45
NT-proBNP	980 pg/mL	< 300

Transthoracic echocardiography showed no abnormalities of the coronary arteries, with a Z-score of <2, confirming the absence of dilation or aneurysm.

The patient was managed according to standard therapeutic recommendations, with intravenous immunoglobulins administered at a dose of 2 g/kg as a single infusion, combined with anti-inflammatory acetylsalicylic acid at 50 mg/kg/day in four divided doses.

The clinical course was rapidly favorable, with resolution of fever within the first 24 hours following infusion. A follow-up laboratory evaluation at 48 hours is summarized in Table 2.

**Table 2:** Laboratory results 48 hours after treatment CRP: C-Reactive Protein; MCV: Mean Corpuscular Volume; MCH: Mean Corpuscular Hemoglobin; ESR: Erythrocyte Sedimentation Rate

Parameter	Result	Evolution
Hemoglobin (Hb)	11.2 g/dL	Stable
MCV	77.1 fL	Stable
MCH	26 pg	Stable
White blood cells (WBC)	11,100/mm³	Decrease
– Neutrophils	3,270/mm³	Marked decrease
– Lymphocytes	6,700/mm³	Relative lymphocytosis
Platelets (PLT)	688,000/mm³	Reactive thrombocytosis
CRP	12 mg/L	Significant decrease
ESR	75 mm (1st h)	Stable (ESR inertia)

**Figure 3:**
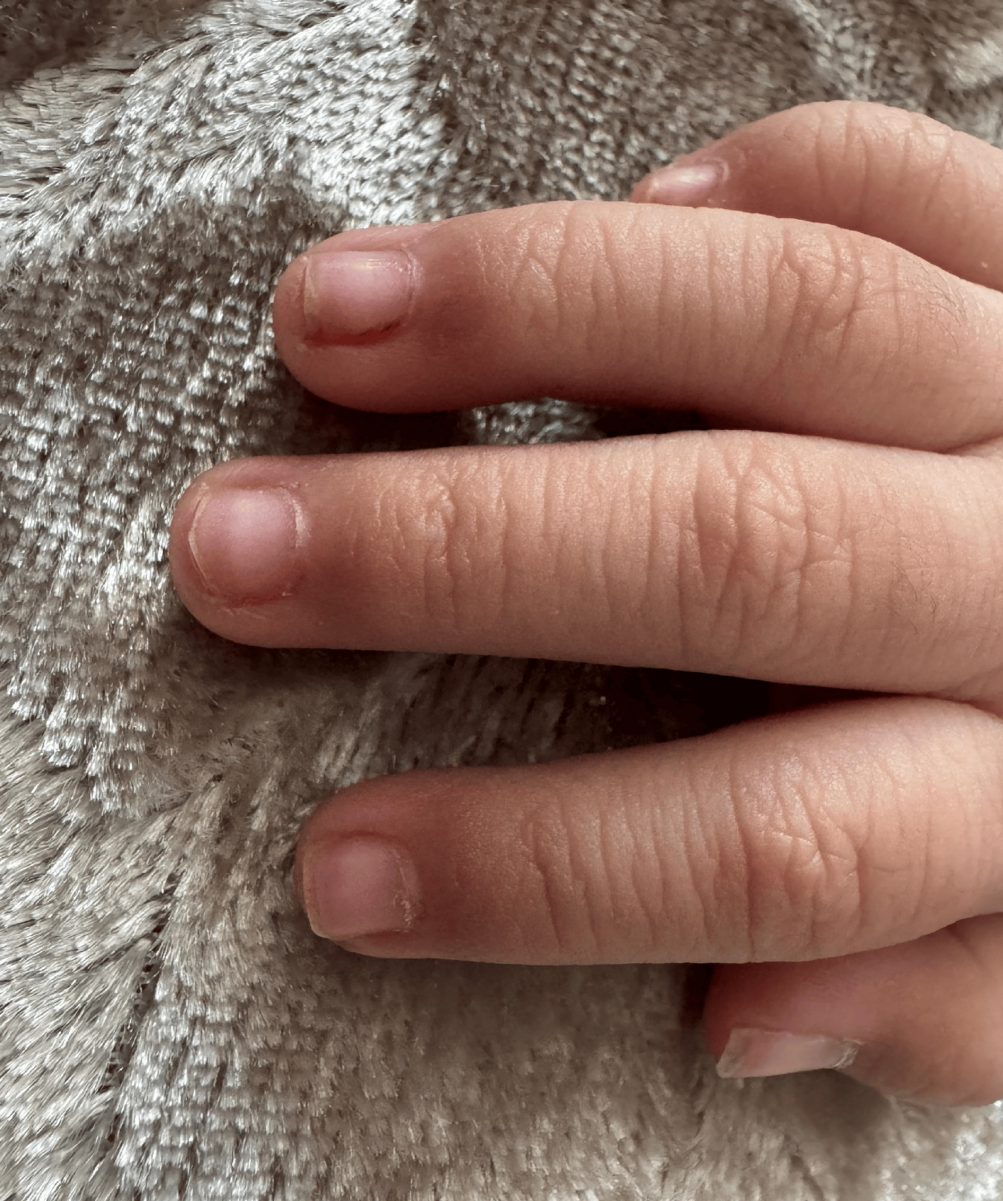
Normal appearance of the fingernails after recovery from Kawasaki disease The previously observed chromonychia has completely resolved, with full restoration of normal nail coloration.

The child was discharged on low-dose aspirin (3-5 mg/kg/day) for antiplatelet therapy, with scheduled echocardiographic follow-up at one and two weeks. Follow-up echocardiography remained normal. Dermatologically, the nail chromonychia gradually faded, disappearing completely after four weeks, without any residual cutaneous manifestations (Figure 3).

## Discussion

KD is an acute inflammatory syndrome that predominantly affects medium- and small-sized arteries, particularly the coronary arteries. Although its exact pathogenesis and etiology remain poorly understood, it is the leading cause of acquired heart disease in children in developed countries [1]. The diagnosis of classic KD is based on the presence of persistent fever for more than five days, associated with at least four of the following five clinical criteria: bilateral non-purulent conjunctivitis, oral mucosal changes (erythema, cheilitis, “strawberry tongue”), extremity changes (erythema, edema, desquamation), polymorphous skin rash, and non-suppurative cervical lymphadenopathy [1,3].

In contrast, in atypical or incomplete forms, diagnosis can be more challenging. The absence of certain classical criteria may delay disease recognition, lead to late treatment initiation, and consequently increase the risk of complications, notably coronary aneurysms. In this context, recognizing unusual but suggestive clinical signs can be valuable. Among such secondary manifestations, nail abnormalities, such as transverse orange-brown chromonychia, may serve as additional diagnostic clues, particularly during the subacute phase [5]. Although infrequent, these nail changes deserve to be known and actively sought, as they may help guide clinicians toward the diagnosis of KD, especially in atypical presentations.

Orange-brown chromonychia is a relatively rare nail manifestation reported during the acute phase of KD [4,6], generally between the fifth and tenth days of fever [7], and occasionally as early as the fourth day, making it a potentially useful early diagnostic marker [8]. This finding was first described in 1992 by Lindsley [4]. The pathophysiological mechanism of chromonychia remains uncertain. It may result from a keratinization disorder of the nail plate or be a direct consequence of vasculitis, particularly due to periungual capillary alterations demonstrated by capillaroscopy in KD patients [9]. The color variation, from orange to brown, may reflect a vascular inflammatory sequence followed by residual pigmentation [4]. According to Pal et al., this discoloration could be related to a dense band of microscopic splinter hemorrhages observed on dermoscopy, followed by persistent pigmentation [8].

These pigmented lines remain stably visible for 7 to 10 days, are more common on the fingers than on the toes, and begin to fade toward the end of the second week of illness [8]. This nail finding has been described in isolated cases. Lindsley reported a prevalence of 15.4% (4 out of 26 cases) among patients with typical KD [10]. In contrast, Mitsuishi et al. observed this anomaly in only 3 of 307 patients (about 1%) [5], whereas Pal et al. reported a much higher prevalence of 72.5% (29 out of 40 cases) in a cohort followed over 3 years [11]. These latter authors even suggested including orange-brown chromonychia as an additional clinical sign in the current diagnostic approach to KD.

Chromonychia has been reported in patients from India, Australia, South Korea, and the United States, and more recently in two children from Mexico and Japan [4]. To our knowledge, no cases have yet been described in Africa. A summary of all cases reported to date is presented in Table 3.

**Table 3:** Reported cases of orange-brown chromonychia in Kawasaki disease in the literature NR: not reported

Author (Year)	Age and Sex	Location	Onset of Lesions	Resolution Time	Kawasaki Disease Type	Associated Anomalies
Lindsley CB [10] 1992	Boy, 18 months	Fingers and toes	NR	After 2 weeks	Typical Kawasaki	None
Lindsley CB [10] 1992	Girl, 2 years	Fingers and toes	NR	After 2 weeks	Typical Kawasaki	Beau’s lines
Lindsley CB [10] 1992	Boy, 10 years	Fingers and toes	NR	After 2 weeks	Typical Kawasaki	None
Lindsley CB [10] 1992	Boy, 8 years	Fingers and toes	NR	After 2 weeks	Typical Kawasaki	None
Pal P & Giri PP [11] 2013	29 patients < 10 years	Fingers and toes	5th to 8th day	After 3 weeks	Typical Kawasaki	Some cases of leukonychia
Chan Ho Na & Sang Ho Youn [12] 2013	Korean girl, 5 years	Fingers and toes	After 10th day	After 14 days	Typical Kawasaki	Periungual desquamation
Raju Thapa & Priyankar Pal [13] 2014	Indian girl, 3 years 4 months	Fingers and toes	5th day	After 6 weeks	Typical Kawasaki	Distal onycholysis, periungual desquamation, transverse leukonychia
Raju Thapa & Priyankar Pal [13] 2014	Indian boy, 2 years 6 months	Fingers and toes	6th day	After 4 weeks	Typical Kawasaki with myocarditis and coronary dilation	Distal onycholysis, partial onychomadesis, transverse leukonychia
James R & Burgner D [7] 2015	Taiwanese-Australian girl, 4 years	Fingers and toes	8th day of illness	After 6 weeks	Typical recurrent Kawasaki (2nd episode)	None
Tessarotto L et al. [14] 2015	Italian girl, 4 years	Fingers and toes	14th day of illness	NR	Typical Kawasaki	None
George AP et al. [15] 2015	American girl, 2 years	Fingers and toes	6th day	NR	Typical Kawasaki	None
Kalasekhar V & Venkatesh C [16] 2015	Boy, 2 years	Fingers and toes	10th day	NR	Typical Kawasaki	Onychomadesis
Yamazaki-Nakashimada et al. [4] 2019	Mexican girl, 2 years	Fingers and toes	2 weeks after	NR	Typical refractory Kawasaki	NR
Yamazaki-Nakashimada et al. [4] 2019	Japanese girl, 2 years	Fingers and toes	9th day of illness	After 2 months	Typical resistant Kawasaki	None
Jindal AK et al. [17] 2020	Boy, 5 years	Fingers and toes	6 weeks	NR	Typical Kawasaki	Onychomadesis
Mitsuishi T et al. [5] 2022	Japanese girl, 30 months	Fingers and toes	14th day of illness	After 3 weeks	Typical Kawasaki	None
Mitsuishi T et al. [5] 2022	Japanese girl, 21 months	Fingers and toes	5th day of illness	After 4 weeks	Typical Kawasaki with coronary dilation	None
Mitsuishi T et al. [5] 2022	Japanese girl, 59 months	Fingers and toes	7th day	After 4 weeks	Typical Kawasaki	None
Kostara M et al. [6] 2023	Greek girl, 6 years	Fingers and toes	10th day	NR	Atypical Kawasaki	None

In our case, chromonychia appeared on the seventh day of fever and progressively disappeared over four weeks, consistent with data from the literature. Published observations show that this manifestation is generally isolated and unrelated to KD severity, thus excluding its use as a severity marker. It most often occurs during the acute phase, although cases with delayed diagnosis have been described. Therefore, its presence, readily visible and easily detected on clinical examination, may provide a relevant diagnostic clue, particularly in supporting the diagnosis of atypical or incomplete forms. Other potential causes of chromonychia should be considered in the differential diagnosis, including infections (fungal or bacterial), trauma, drug- or topical-induced nail discoloration, and systemic disorders affecting hepatic or renal function. Specific reports have documented chromonychia, particularly melanonychia, as a side effect of certain antineoplastic agents such as doxorubicin, cyclophosphamide, and vincristine. It may also arise following burns, exposure to elemental iron, treatment with angiotensin II receptor antagonists, or the use of nail hardeners, and has been associated with systemic conditions including systemic lupus erythematosus, hyperbilirubinemia, systemic arthritis, and hemophagocytic lymphohistiocytosis [11]. In our patient, these alternative etiologies were excluded based on the absence of recent drug or topical exposure, lack of clinical or laboratory evidence of systemic disease, and absence of local signs of infection, thereby supporting the association of chromonychia with KD.

Beyond chromonychia, other nail changes have been described in KD. The most frequently reported are transverse leukonychia and Beau’s lines [4]. Rarer findings have also been documented. Ciastko reported a case of onychomadesis occurring six weeks after a febrile episode of KD [18]. Furthermore, pincer nail deformities have also been observed in this disease [12]. These changes most often occur during the chronic phase and resolve spontaneously.

## Conclusions

Orange-brown chromonychia, although rarely reported, represents an intriguing nail manifestation of Kawasaki disease (KD). Its clinical recognition, still marginal, could serve as an accessible early marker, particularly in atypical or incomplete forms of the disease where diagnosis may be delayed. Our observation highlights the value of nail examination in the assessment of febrile patients with suggestive signs of KD. While the specificity and diagnostic value of this anomaly remain to be determined, the growing number of clinical cases reported in the literature suggests it deserves consideration in the diagnostic process. Prospective studies involving larger cohorts are needed to better define its prevalence, pathophysiological mechanism, and added value in clinical diagnosis.
